# Slipping through: mobility’s influence on infectious disease risks for justice-involved women in Canada

**DOI:** 10.1186/s40352-021-00157-3

**Published:** 2021-11-29

**Authors:** Susie Taylor, Margaret Haworth-Brockman, Yoav Keynan

**Affiliations:** National Collaborating Centre for Infectious Diseases, Rady Faculty of Health Sciences, University of Manitoba, Room L332A, Basic Medical Sciences Building, 745 Bannatyne Ave, Winnipeg, Manitoba R3E 0T5 Canada

**Keywords:** Migration, Incarceration, Sexually transmitted and blood-borne infections, Women, Canada

## Abstract

**Background:**

The relationship between incarceration and women’s vulnerability to sexually transmitted and blood-borne infections (STBBI) is understudied in Canada, despite numerous studies showing that justice-involved women experience very high rates of infection. Justice-involved women in Canada are highly mobile, as a result of high rates of incarceration and extremely short sentences. From a public health perspective, it is productive to understand how the mobility of justice-involved women shapes their vulnerability to STBBI.

**Results:**

This narrative review demonstrates that mobility between incarceration facilities and communities drives sexually transmitted and blood-borne disease risk for justice-involved women in Canada. Associations and interactions between epidemics of gender-based and intimate partner violence, substance use, and STBBIs shape the experiences of justice-involved women in Canada. In correctional facilities, the pre-existing vulnerability of justice-involved women is compounded by a lack of comprehensive STBBI care and limited harm reduction services. On release, unstable housing, disruptions to social support networks, interruptions in medical care, and relapse to or continuation of substance use, significantly increase individual disease risk and the likelihood of community transmission. High rates of incarceration for short periods perpetuate this cycle and complicate the delivery of healthcare.

**Conclusions:**

The review provides evidence of the need for stronger gender-transformative public health planning and responses for incarcerated women, in both federal and provincial corrections settings in Canada. A supportive, evidence-based approach to STBBI identification and treatment for incarcerated women - one that that removes stigma, maintains privacy and improves access, combined with structural policies to prevent incarceration - could decrease STBBI incidence and interrupt the cycle of incarceration and poor health outcomes. A coordinated and accountable program of reintegration that facilitates continuity of public health interventions for STBBI, as well as safe housing, harm reduction and other supports, can improve outcomes as well. Lastly, metrics to measure performance of STBBI management during incarceration and upon release would help to identify gaps and improve outcomes for justice-involved women in the Canadian context.

## Background

Mobility is increasingly recognized as a public health priority, both as a factor in the success of public health initiatives and as an important social determinant of health (Tulloch et al., 2016). At an individual level, human mobility can provide new pathways to healthcare, but it can also result in changes in exposure to infectious diseases, interruptions in care, psychosocial stressors, socioeconomic consequences, and linguistic, social and administrative barriers to health services (International Organization for Migration, 2017; Tulloch et al., 2016). To achieve the UN 2030 Sustainable Development Goals – which emphasize universality and “no one left behind” – it is necessary to gather data on, and develop policies that address, the complex interrelationships between health and human mobility (International Organization for Migration, 2017, 2018; Tulloch et al., 2016; Wickramage et al., 2018).

From a public health perspective, it is productive to think about incarceration as a form of mobility. Like other migrants, justice-involved women in Canada are highly mobile (Statistics Canada, n.d.; Stewart, 2019); they also experience distinct health vulnerabilities that differ from the general population. These include high rates of mental health diagnoses and psychiatric hospital admissions, head injuries, substance use disorders, and communicable diseases (Correctional Service Canada, 2018; Kouyoumdjian et al., 2016; Martin et al., 2012; Nolan & Stewart, 2014).

Justice-involved women are also vulnerable to sexually transmitted and blood-borne infections (STBBI), with infection rates significantly higher than either the general population or the incarcerated male population (Taylor & Neufeld, 2021). The relationship between incarceration and women’s vulnerability to STBBI is understudied in Canada, despite numerous studies showing very high rates of infection in justice-involved women (Gratrix, Smyczek, et al., 2019; Kouyoumdjian et al., 2012; Zakaria et al., 2010).

In the following, “justice-involved women” refers to people assigned female at birth who have spent time in a correctional facility in Canada; other forms of involvement with the judicial system are excluded. Federal corrections in Canada placed people according to assigned sex at birth until 2017, and at present, only the Yukon (2017), British Columbia (BC) (2016), and Ontario (2012) have specific policies for placement of transgender inmates. There are very few data collected on the experiences of non-binary and transgender incarcerated people in Canada, and most literature reflects assigned sex at birth. This paper will include the carceral experiences of transgender women where literature is available, using the term “trans women”.

In Canada, the current epidemics of STBBI have been described as a syndemic: they share underlying determinants, co-infections are common, and these infections interact to heighten health consequences (Public Health Agency of Canada, 2020b). Evidence suggests that incarceration is an underlying determinant: in particular, high rates of short-term incarceration are linked to increased STBBI transmission in both correctional settings and communities (Taylor & Neufeld, 2021). Gender and racial disparities in Canadian incarceration rates, and recognized gender disparities in correctional healthcare (Socías et al., 2015), illustrate the urgent need for new public health approaches that mitigate inequities (Pederson et al., 2015). In this paper, we review the available evidence to determine how movement in and out of incarceration affects vulnerability to infectious diseases in justice-involved women, suggesting that public health consider this mobility as a driver of STBBI transmission. We provide evidence of the need for equity-based, woman-centered, and trauma-informed approaches to public health planning and responses for incarcerated women, in both federal and provincial corrections settings.

## Methods

We conducted a narrative review (Ferrari, 2015; Rother, 2007) to determine the scope of current literature from a variety of sources. Our goal was to identify: STBBI burden in justice-involved women in Canada; mobility related to incarceration in this group; risk factors and social determinants of STBBI in this group; and healthcare in Canadian correctional facilities. We searched PubMed, Scopus, the database “Research at the Correctional Service of Canada”, and the Public Health Agency of Canada’s “Reports and Publications on Sexually Transmitted Infections in Canada”. The analysis was carried out by the National Collaborating Centre for Infectious Diseases, under the project streams “HIV/STBBI Prevention and Control” and “Migration and Mobility”.

## Results

### Women, incarceration and mobility

In Canada, very short periods of incarceration and rapid re-entry to community, as well as high recidivism, mean that there is significant mobility of justice-involved women. Women serve shorter sentences and return to community sooner than do men (Statistics Canada, n.d.). In 2018–19, 57.6% of women in federal and provincial/territorial facilities served their time in 1 month or less, and 9.2% of women served their time in one to three months. Mobility in and out of incarceration is increased further by recidivism (for women in federal corrections, recorded rates are between 12 and 20%) and other unrecorded forms of re-incarceration, such as entrances to remand that do not end in a conviction (Stewart et al., 2019). Frequent movement in and out of incarceration facilities for short periods of time is a significant driver of sexually transmitted and blood-borne infection (STBBI) risk and transmission in Canada.

### Women, incarceration and STBBIs

Globally, incarcerated people have higher rates of infectious diseases, including STBBIs, than general populations (Courtemanche et al., 2018; Flanigan et al., 2009; Flanigan & Beckwith, 2011; Kouyoumdjian et al., 2012; Stone et al., 2018; Taylor & Neufeld, 2021; Wiehe et al., 2015; Wirtz et al., 2018). Prevalence rates of Human Immunodeficiency Virus (HIV), Hepatitis C (HCV), and Hepatitis B (HBV), (as well as Tuberculosis (TB)) are significantly higher in correctional populations than in the general population, particularly for people who inject drugs (PWID) (Taylor & Neufeld, 2021). Prevalence of other STBBI, including chlamydia, gonorrhea, and syphilis is also higher for incarcerated people globally (Taylor & Neufeld, 2021).

STBBI rates are far higher for incarcerated people than for the general population in Canada (Taylor & Neufeld, 2021); they are especially high for incarcerated women, who are more likely to be impacted by the “SAVA syndemic”, a term describing interrelated and mutually amplifying health conditions related to violence, substance use, and HIV (Flanigan & Beckwith, 2011; Gilbert et al., 2015; Stoltey et al., 2015). Over half of incarcerated people in Canada have childhood histories of abuse, and incarcerated women are more likely than men to have been neglected or sexually abused in childhood (Bodkin et al., 2019). Trauma amongst those who have experienced childhood abuse and/or gender-based violence has been associated with high-risk sexual behaviours as well as mental health and substance use disorders (Gottlieb & Mahabir, 2021). Prevalence of mental health disorders is very high for incarcerated women: the Correctional Service of Canada (CSC) estimates that more than three-quarters of justice-involved women had a lifetime or current mental disorder, and two-thirds reported symptoms of a co-occurring mental disorder with alcohol/substance use (Correctional Service of Canada, 2018). Substance use in community settings can increase the risk of acquiring STBBIs, even for non-injection drugs like methamphetamines, because of an association with high-risk behaviours (Public Health Agency of Canada, 2020b, pg. 67). For Canadian women, recent incarceration is associated with current sex work, injection drug use, unstable housing and lower annual income, suboptimal antiretroviral therapy adherence, and Indigenous ancestry (Gormley et al., 2020). Cumulative disadvantage shapes the life experiences of most justice-involved women, and heightens their vulnerability to STBBI.

Women in Canadian federal corrections have a 4.5 time greater incidence of STBBI diagnosis since admission than men (Zakaria et al., 2010). Among federally incarcerated women, 7.9% self-reported being HIV-positive, compared with 0.21% in the general population during the same time period (Public Health Agency of Canada, 2015). Among Aboriginal (sic) women who were federally incarcerated, 11.7% reported HIV positivity (Zakaria et al., 2010). Female STBBI rates after admission to federal corrections, compared to female rates in the general population, were: 1230 vs. 257/100,000 for chlamydia, 980 vs. 19/100,000 for gonorrhea, and 250 vs. 2/100,000 for syphilis (Zakaria et al., 2010). Given the rapidly rising syphilis incidence in Canada (Choudhri et al., 2018; Public Health Agency of Canada, 2020b), it is likely the latter rates are significantly higher now.

Provincial incarceration periods are typically shorter, with arguably more population mobility. There is more variation in STBBI rates in provincial and territorial corrections, and more heterogeneous approaches to testing, but high prevalence rates, and gender differences within those rates, persist (Courtemanche et al., 2018; Kouyoumdjian et al., 2012). In provincial facilities, approximately 30% of women are infected with hepatitis C virus (HCV), double the infection rates for men (Kouyoumdjian et al., 2016). Although it is more difficult to find data on chlamydia, gonorrhea, and syphilis in provincial corrections populations, there are indications that women are disproportionately affected. For example, in three provincial correctional facilities in Alberta, a study of STBBI testing found syphilis prevalence of 5.4% in female facilities (Public Health Agency of Canada, 2020b).

International and Canadian research suggest that the risk of both incarceration and STBBIs is high for transgender women (Brömdal et al., 2019; Poteat et al., 2018; Socías et al., 2015). A study among sex workers in BC found that being LGBT*2S was correlated with incarceration; the correlation was strongest for transgender sex workers (Socías et al., 2015). Internationally, incarcerated transgender women experience high rates of violent victimization, including rape and coerced sex, which significantly increases their risk of STBBI and trauma that may lead to high risk substance use (Brömdal et al., 2019; Poteat et al., 2018). Despite these concerning indicators, there is little scholarship that describes the experiences of incarcerated transgender people or their vulnerability to STBBI in the correctional environment (Poteat et al., 2018; Wirtz et al., 2018): there is even less in a Canadian context.

## Discussion

### Incarceration, mobility, and community transmission

The well-being and health of families, neighborhoods and communities depend on the health of incarcerated people (Flanigan & Beckwith, 2011). Communities with higher incarceration rates have elevated STBBI rates, and high rates of turnover in incarceration facilities appear to perpetuate infectious disease transmission in communities (Taylor & Neufeld, 2021). Policies and practices that provide STBBI prevention, testing, and care services in correctional facilities - as well as continuity of medical care after release - have the potential to improve public health, reduce STBBI prevalence, reduce recognized gendered and racialized healthcare inequalities in corrections (Socías et al., 2015), and lower recidivism (Wang et al., 2012). Researchers have emphasized the value of STBBI testing and management in correctional facilities, and expressed concern that opportunities to interrupt transmission are not being used (Flanigan & Beckwith, 2011; Kouyoumdjian et al., 2012, 2016; Morrow et al., 2009; Pathela, 2014).

Movement between incarceration facilities and community settings appears to independently increase the risk of acquiring STBBI and/or failing to adhere to treatment. Studies in BC found that incarceration is independently associated with: a greater than 2.5 fold risk of HIV seroconversion in people who inject drugs (Werb et al., 2008); increased odds of non-adherence to HIV treatment (Palepu et al., 2004); and lower to non-existent viral suppression for cis and trans women living with HIV (Erickson et al., 2020). The authors of the latter study concluded that while we often see incarceration as a missed opportunity for engagement in HIV or STBBI care, incarceration may itself be associated with interruptions in, and/or difficulty adhering to, HIV treatment (Erickson et al., 2020). The negative effects of incarceration may disproportionately affect women’s treatment outcomes: a systematic global literature review found that previously incarcerated women had lower rates of engagement in care, anti-retroviral adherence, and HIV viral suppression after incarceration than men, although their outcomes while in corrections were similar (Erickson et al., 2019). This suggests that incarceration, particularly for short periods, can be a barrier to STBBI treatment adherence and its associated positive outcomes, especially for women.

### STBBI healthcare for justice-involved women

There are several reasons why incarceration is so disruptive to STBBI detection and treatment. Although international minimum standards specify that incarcerated people should have the same healthcare as they would within their community, and this is a statutory requirement for federal institutions (The John Howard Society of Ontario, 2017; United Nations, 2015), the structure of correctional health services in Canada means that transitions in and out of incarceration can interrupt necessary STBBI services. At the federal level, health services are provided by CSC rather than a health agency, and healthcare is governed by various Commissioner’s Directives, in particular the Commissioner’s Directive on Health Services. At the provincial/territorial level, health services for incarcerated people are provided by either the provincial health ministry (as in Nova Scotia, Alberta, and British Columbia) or more commonly, the provincial corrections ministry (The John Howard Society of Ontario, 2017). As a result, in many correctional facilities, health care is delivered by a parallel system, which can have implications for continuity of care, availability of services, and record-keeping during transitions between the institution and community (The John Howard Society of Ontario, 2017). Many developed countries, including Norway, France, and the UK, have transitioned from a parallel model to an integrated health care system, based on a directive from the Committee of Ministers of the Council of Europe. Although challenging in a federated system, this approach is worth considering in Canada.

Incarcerated Canadians use healthcare more than the general population, matched for age, both while in corrections and after release (Bernier & MacLellan, 2011; Bruggman, 2012; Kouyoumdjian et al., 2018; Kouyoumdjian & McIsaac, 2015). Despite high rates of use, surveys in Ontario and Nova Scotia showed that justice-involved women felt that they could not get the care they needed while incarcerated (Bernier & MacLellan, 2011; Kouyoumdjian et al., 2018). In both studies, women reported poorer health status than men, more frequent use of health services, and greater dissatisfaction with the healthcare they received (Bernier & MacLellan, 2011; Kouyoumdjian et al., 2018). For example, 60% of respondents in Nova Scotia reported receiving healthcare only “some of the time,” and an additional 27% said they were “never” able to use health services when they needed them (Bernier & MacLellan, 2011). Female participants were more than twice as likely as men to report that their health worsened during incarceration (Bernier & MacLellan, 2011).

In a high prevalence environment for communicable diseases, early identification and management (including contact tracing) are essential for interrupting transmission. However, evidence shows that this is not happening in Canadian incarceration facilities (Gormley et al., 2020; Kouyoumdjian et al., 2016; Werb et al., 2008). For example, shortfalls in HCV care in corrections have been identified, related to interruption of provincial drug benefit programs, poor follow-up during transfer and release, short periods of incarceration, a lack of confidentiality when obtaining medical care, and stigma among incarcerated people and staff (The Canadian Network on Hepatitis C Blueprint Writing Committee and Working Groups, 2019). Most incarcerated women in Canada are of typical childbearing age (Socías et al., 2015); detection of syphilis in this population is of particular concern given the high prevalence of infection and risk of vertical transmission with congenital infection. Syphilis is complicated to manage, particularly in correctional environments with frequent population movement, since it requires a series of three injections. There are successful examples described in literature (for ex., Harmon et al., 2020), but not in a Canadian context. Interruptions in treatment and serological follow-up can contribute to ongoing transmission and sequelae for the individual (such as neurosyphilis or involvement of the cardiovascular system); as such, it is necessary to understand how the mobility of justice-involved women affects the consistency of their treatment.

In a study of opt-out testing in Alberta provincial corrections, estimated testing rates for STBBI at admissions ranged from 4.8 to 16.1% (Gratrix, Smyczek, et al., 2019). The authors noted the “vast majority” of incarcerated individuals are not screened or are only screened for certain STBBI (Gratrix, Smyczek, et al., 2019, pg. 273), despite the ways in which STBBI interact to increase vulnerability. (For example, untreated infections such as trichomonas, chlamydia, gonorrhea, and mycoplasma genitalia infections have negative sequelae, particularly for women, that can increase risk of HIV transmission (Public Health Agency of Canada, 2020a)). The study found that opt-out testing at point of admission was feasible, although uptake was limited by factors such as “inability to consent”, “operational limitations”, and “refusal of consent” (Gratrix, Payne, et al., 2019). Notably, 35.3% of those refusing consent did so because they had “no perceived risk”; however, positivity rates for incarcerated women were 26.1% for gonorrhea and 21.8% for chlamydia (Gratrix, Payne, et al., 2019). Federal correctional guidelines dictate that incarcerated people should be offered voluntary screening and risk-based testing for STDs (sic) on admission (Correctional Service of Canada, 2019). Nonetheless, it is likely that a lack of comprehensive STBBI testing in both federal and provincial corrections contributes to high rates of infectious disease transmission.

Other factors include a lack of timely follow-up and contact notification for positive results, information and data platforms that are separate from provincial health databases, and insufficient communication between correctional facilities and community healthcare settings during arrest, incarceration, or release. For example, federal reintegration programs address issues such as housing, education and social programs, but do not explicitly address the handover of public health interventions (Correctional Service of Canada, 2019). Crowding in limited shared space facilitates contact between incarcerated people, creating opportunities for high risk sexual behaviours or needle sharing. Delays in medical care due to security procedures can further increase transmission of infections within incarceration facilities (Flanigan & Beckwith, 2011). Interruption of medical care during admission and on release can also have deleterious effects on infectious disease treatment. However, there are successful examples of STBBI health care provision in international correctional facilities, even those that are overcrowded and have frequent movement in and out of facilities. Flanigan et al. describe a collaboration between Rhode Island incarceration facilities and Brown University’s Alpert Medical School to offer opt-out HIV testing on admission; rapid testing within the facility; clinical services after diagnosis; and a facilitated release program in which medical care providers, social and outreach workers act as a case management team to ensure consistency of medical care and linkage to community-based services (Flanigan et al., 2009).

### Harm reduction services in Canadian correctional facilities

Mobility in and out of incarceration can also expose justice-involved women to risks arising from substance use or sex that take place in facilities. Substance use is prevalent in Canadian corrections, despite efforts to prevent use and reduce availability of illicit drugs (Correctional Service of Canada, 2019; Correctional Service of Canada, 2018). Harm reduction policies and practices for substance use vary in Canadian provincial, territorial, and federal facilities, despite evidence supporting the value of such programs (Glauser, 2013). Correctional legislation and policies often have a zero tolerance approach to drug possession, and possession of needles, outside of certain circumstances (Office of the Correctional Investigator, 2019). In 2018, CSC began a needle exchange program, in part to reduce the spread of diseases like HIV and HCV. As of 2019, the program was available in 9 of 53 federal institutions, although participation in the program is limited by disciplinary measures for participants found in possession of illegal drugs (Correctional Service of Canada, 2019; Office of the Correctional Investigator, 2019). Data are sparse, but needle exchange programs do not appear to be available in the majority of provincial and territorial facilities. The discrepancy between best practices and existing policies is due to concerns that harm reduction supplies carry security risks, and that harm reduction policies will increase substance use among incarcerated people. However, when needle exchange programs have been implemented, these issues have not materialized (Werb et al., 2008). Safe sex supplies such as dental dams, condoms, and lubrication are available in most Canadian correctional facilities, including mixed and women’s-only corrections. However, incarcerated people often need to ask for these supplies, which contravenes international guidelines (Office of the Correctional Investigator, 2019). There is American evidence showing the importance of both privacy and a stigma-free environment in encouraging use of harm reduction services in incarceration (Erickson et al., 2020); it is probable that lack of privacy and stigma around harm reduction are barriers to use in Canadian correctional facilities.

### Return to community and intersecting vulnerabilities

The limitations of the correctional system in interrupting STBBI transmission are compounded by the intersecting vulnerabilities faced by justice-involved women when they return to community. At the individual level, incarceration “leads to economic vulnerability, cumulative disadvantage, and limited access to educational opportunities and social and risk reduction services” (Stockman & Strathdee, 2010, pg. 5): movement in and out of incarceration compounds these effects, by negatively affecting housing, employment, and social relationships. Mobility in and out of incarceration facilities can also increase vulnerability to the SAVA syndemic. American studies have shown that people on parole or in supervision “may be more likely to engage in relationships with risky sex partners” (Green et al., 2012, pg. 424)”. Cis and trans women parolees in Canada and internationally are more likely to engage in sexual relationships for drugs, housing, or money after incarceration (Brömdal et al., 2019; Gilbert et al., 2015; Gormley et al., 2020). Disruptions to family relationships as a result of incarceration can disproportionately affect women who are mothers. In 2003, an estimated 20,000 Canadian children were separated from their mothers due to incarceration; the figure is likely higher now, as female incarceration has increased significantly since that time (Janssen et al., 2017; Martin et al., 2012). Traumatic separations increase the likelihood of re-incarceration, while the existence of a better relationship between mothers and their children makes a successful transition more likely (Martin et al., 2012). Participatory research with incarcerated women in BC identified improved family relationships as “essential for their successful reintegration into society following their release from prison” (Martin et al., 2012, pg. 506). Incarceration, particularly for short periods, disrupts the family lives, support systems, and living conditions of already-vulnerable women and in so doing may increase the likelihood that they will engage in high risk behaviours.

Justice-involved women returning to community need social supports for themselves and their families, healthcare, housing, and employment (Woods et al., 2013). However, incarceration can be a significant barrier to these supports, particularly obtaining housing (which often determines ability to use other social services) and employment (Smith et al., 2019). Housing instability is associated with substance use, depression, HIV positive status, and, for women, mortality (Martin et al., 2012; Socías et al., 2015). A BC survey found that homelessness was a factor in the re-incarceration of 56% of women (Martin et al., 2012). Homelessness can also impel sex workers to work in public spaces, increasing their sexual, drug-related, and violence risks, and thereby their risk of STBBI (Socías et al., 2015). Sex work in public spaces is also correlated with incarceration because of Canada’s enforcement based approach to prostitution (Socías et al., 2015). This indicates that those sex workers who are most at risk of contracting STBBI are also at higher risk of incarceration, furthering the potential for individual infection and community transmission.

The physical and mental health status of formerly incarcerated people affects employment, family relationships, and finances; poor health has been linked to re-entry failures and re-incarceration (Link et al., 2019). However, a “disconnect between health care services in correctional facilities and those in the community” further compounds the health risks faced by justice-involved women in Canada (McLeod & Martin, 2018, pg.274) An integrated model of healthcare delivery, one that is governed by the provincial health ministry, and that has coordinated and accountable links to community organizations, has greater capacity to facilitate the transition from incarceration to community (McLeod & Martin, 2018; Smith et al., 2019), potentially reducing the harms of this mobility. Use of enhanced or integrated primary health care has been linked to reduced recidivism and reduced re-incarceration time (Wang et al., 2019), and may further mitigate the negative effects of incarceration. Coordinated interventions to help formerly incarcerated Canadians to navigate services in the community on release can have a positive effect on their health and familial relationships, while also reducing recidivism (Fahmy et al., 2018). Although data specific to women are not available, these interventions could interrupt STBBI transmission by providing easier pathways to care, decreasing stigma associated with disease-specific programs, decreasing risky behaviours, and reducing movement between corrections and community. Improved data flow from corrections to community may also improve treatment completion and follow-up for syphilis, and encourage continued engagement in HIV and HCV treatment programs.

## Conclusions

Even a cursory glance at STBBI rates shows that there is a concerning link between incarceration and STBBI burden and management. Associations and interactions between epidemics of gender-based and intimate partner violence, substance use, and STBBIs shape the experiences of justice-involved women in Canada. In correctional facilities, the pre-existing vulnerability of justice-involved women – due to experiences of childhood abuse and/or gender-based violence, high rates of substance use, and high risk sexual behaviours – is compounded by a lack of comprehensive STBBI care and limited harm reduction services. On release, justice-involved women are further disadvantaged by lower rates of employment, higher rates of substance use, gender-based violence, sexual exploitation, and (often) being primary caregiver. Unstable housing, disruptions to social support networks, interruptions in medical care, and relapse to or continuation of substance use, further increase the risks of infection and community transmission. Short periods of incarceration and high recidivism perpetuate this cycle, while complicating delivery of healthcare. Seen as a whole, justice-involved women’s mobility in and out of correctional facilities is a significant driver of STBBI epidemics in Canada.

Despite this fact, there are currently no available standards or recommendations for STBBI public health and healthcare in correctional environments in Canada, nor are there guidelines on the handover of public health interventions at incarceration or on release. There is also a lack of regularly collected, publicly available data regarding STBBIs in federal and provincial/territorial correctional facilities, making it more difficult to develop evidence-based interventions and policies. The development of public health interventions that recognize the mobility of justice-involved women– and its deleterious effects – could reduce healthcare inequalities, thereby improving public health and many lives. As others have argued in different contexts, a gender transformative approach to public health for justice-involved women is necessary to address the root causes of health inequities (Bloom, 2005). This involves “address[ing] gender as an element of a social system and structure” (Pederson et al., 2015, pg. 146), centering justice-involved women’s life experiences, rights, preferences, and roles in health promotion efforts.

A supportive, evidence-based approach to STBBI identification and treatment for incarcerated women that removes stigma, maintains privacy and improves access, combined with structural policies to prevent incarceration, could decrease STBBI incidence and interrupt the cycle of incarceration and poor health outcomes. A coordinated and accountable program of reintegration that facilitates continuity of public health interventions for STBBI, safe housing, harm reduction, and addresses barriers to successful community transition - in particular ensuring basic subsistence needs are met - can improve outcomes as well (McLeod & Martin, 2018; Pederson et al., 2015; Smith et al., 2019). Following the principles of gender transformative health promotion, these interventions should be woman-centered, trauma-informed, and strength-based. Lastly, metrics to measure performance of STBBI management during incarceration and upon release would help to identify gaps and improve outcomes for justice-involved women in the Canadian context.

## Data Availability

Not applicable. No datasets were generated or analysed during the current study.

## Abbreviations

AIDS: Acquired immunodeficiency syndrome
BC: British Columbia
CSC: The Correctional Service of Canada
HBV: Hepatitis B Virus
HCV: Hepatitis C Virus
HIV: Human Immunodeficiency Virus
LGBT*2S: Lesbian, gay, bisexual, transgender, Two-Spirited, inclusive of other gender or sexual variants
PWID: People who inject drugs
SAVA syndemic: Substance abuse, violence and AIDS syndemic
STBBI: Sexually transmitted and blood-borne infection
STD: Sexually transmitted disease
TB: Tuberculosis
